# Rapidity of the Multiplication of Bacteria

**Published:** 1889-01

**Authors:** 


### RAPIDITY OF THE MULTIPLICATION OF BACTERIA.

Small as they are, bacteria are by no means insignificant. Like aphides and other small insect pests, they possess the power of multiplying with great rapidity: but they far exceed their most active competitors in this respect. Cohn has calculated that a species undergoing fission once an hour,—which is not too high an estimate for some species,—under favorable circumstances might count nearly seventeen million offspring at the end of twenty-four hours, while in about a week the number could be represented only by the use of fifty-one figures, so that it is practically meaningless to the ordinary mind; but to give some means of comparison he calculates the space that these microscopic beings would occupy if each were about twice the size of that I have spoken of, and finds that in five the progeny of a single cell if all survived and were equally prolific, would occupy nine hundred and twenty-eight million cubic miles, the volume of the ocean. Of course their reproduction is checked greatly by unfavorable conditions and by their enemies; but what wonder if the germs of putrefaction and disease are well-nigh omnipresent?

Though bacteria are exceedingly minute and simple beings, we have seen that their power of multiplication, if unchecked, is incomprehensibly great. Their influence on higher organisms is also entirely disproportionate to their size. Containing no assimilating pigment, they resemble other fungi, as well as animals, in being dependent upon organic matter for their nutrition; hence they must live either as saprophytes, utilizing the substance of dead animals or plants, or as parasites, obtaining their food from living beings—often at the expense of the health or life of the latter.—*Medical Register.*

